# Reference ranges of fetal mandible measurements: Inferior facial angle, jaw index, mandible width/maxilla width ratio and mandible length in Thai fetuses at 15 to 23 weeks of gestation

**DOI:** 10.1371/journal.pone.0269095

**Published:** 2022-06-01

**Authors:** Pichaya Booncharoen, Rapphon Sawaddisan, Chitkasaem Suwanrath, Alan Geater

**Affiliations:** 1 Department of Obstetrics and Gynecology, Faculty of Medicine, Prince of Songkla University, Songkhla, Thailand; 2 Epidemiology Unit, Faculty of Medicine, Prince of Songkla University, Songkhla, Thailand; Universita degli Studi dell’Insubria, ITALY

## Abstract

**Objectives:**

To determine the normal distribution of 1) inferior facial angles (IFA), 2) jaw index, 3) mandible width/maxilla width ratio (MD/MX ratio), and 4) mandible length (ML) in second trimester Thai fetuses.

**Methods:**

A prospective study was performed between April 1 and October 31, 2020, at the Maternal-Fetal Medicine Unit of Songklanagarind Hospital. Transabdominal ultrasonography was performed on Thai singleton pregnant women at 15^0/7^ to 23^6/7^ weeks of gestation to measure IFA, jaw index, MD/MX ratio and ML. All women received standard antenatal care and were followed up until delivery. The exclusion criteria were multifetal gestation, congenital anomaly, chromosomal abnormality, fetal growth restriction, abnormal amniotic fluid volume, suspected abnormality of fetal mandible, maxilla or jaws based on the proposed criteria from previous studies and suspected neonatal structural or genetic abnormalities postnatally. Quantile regression was used to estimate changes in the median, 5th and 95th percentiles of each parameter across gestational ages and to generate formulas for predicting the 5th percentile value for each parameter.

**Results:**

The results of 291 women were analyzed. Scatter plots and reference ranges of each parameter were generated. IFA, jaw index and ML values significantly increased, while the MD/MX ratio value significantly decreased, with increasing gestational age. The formulas calculated for predicting the 5th percentile value for each parameter were IFA = 55.12 + 0.045*(GA in days—136) jaw index = 37.272 + 0.01693*(GA in days—136) MD/MX ratio = exp(0.027–0.00110*(GA in days—136)) ML = 20.83 + 0.243*(GA in days—136).

**Conclusions:**

The reference ranges and formulas to calculate the 5th percentile values of mandible parameters in Thai fetuses were developed.

**Trial registration:**

This study has been reviewed and approved by the Thai Clinical Trials Registry with identification number TCTR20210602003.

## Introduction

Detection of fetal abnormalities before birth leads to meticulous neonatal care and reduction of neonatal death [1, 2]. Evaluation of the fetal mandible is included in the standard sonographic second trimester anatomical scan; however, currently there are no clear criteria to define ‘abnormal’ in the areas of fetal mandible scan [3, 4].

There are two main abnormalities of mandible development. The first is micrognathia, which refers to small size of mandible. The second is retrognathia, which refers to an abnormally posteriorly located mandible compared to the maxilla [3]. These rare conditions occur in 1 per 1,500 livebirths worldwide [5]. Newborns with these conditions have increased risks of developing upper airway obstruction or swallowing problems during their immediate postnatal period [6–8]. Some of these neonates will need respiratory support, emergency postnatal airway management and/or long-term airway/swallowing management [8–10]. Abnormal mandible development is also associated with genetic defects, for instance, Pierre-Robin sequence, Treacher-Collin syndrome, branchio-oculofacial syndrome, cri-du-chat syndrome, trisomy 13 and trisomy 18 [4, 11, 12].

Previously, prenatal diagnosis of fetal micrognathia and retrognathia was mainly based on the ultrasonographer’s judgement [3, 11]. There have been ongoing attempts in recent years to establish specific criteria for diagnosing these conditions, based around 4 main parameters, which were 1) inferior facial angles (IFA) [13, 14], 2) jaw index [15], 3) mandible width/maxilla width ratio (MD/MX ratio) [13], and 4) mandible length (ML) [16–19].

Studies done in the United States, France, Italy, England, Germany, China and Singapore during 1991–2019 have found differences of facial biometrics between various ethnicities [13–19]. In the absence of studies evaluating the normal distribution of these four main parameters in Thailand, we aimed to evaluate the normal distribution of 1) inferior facial angles (IFA), 2) jaw index, 3) mandible width/maxilla width ratio (MD/MX ratio), and 4) mandible length (ML) in the second trimester of Thai ethnic fetuses.

## Materials and methods

**Fig 1:**
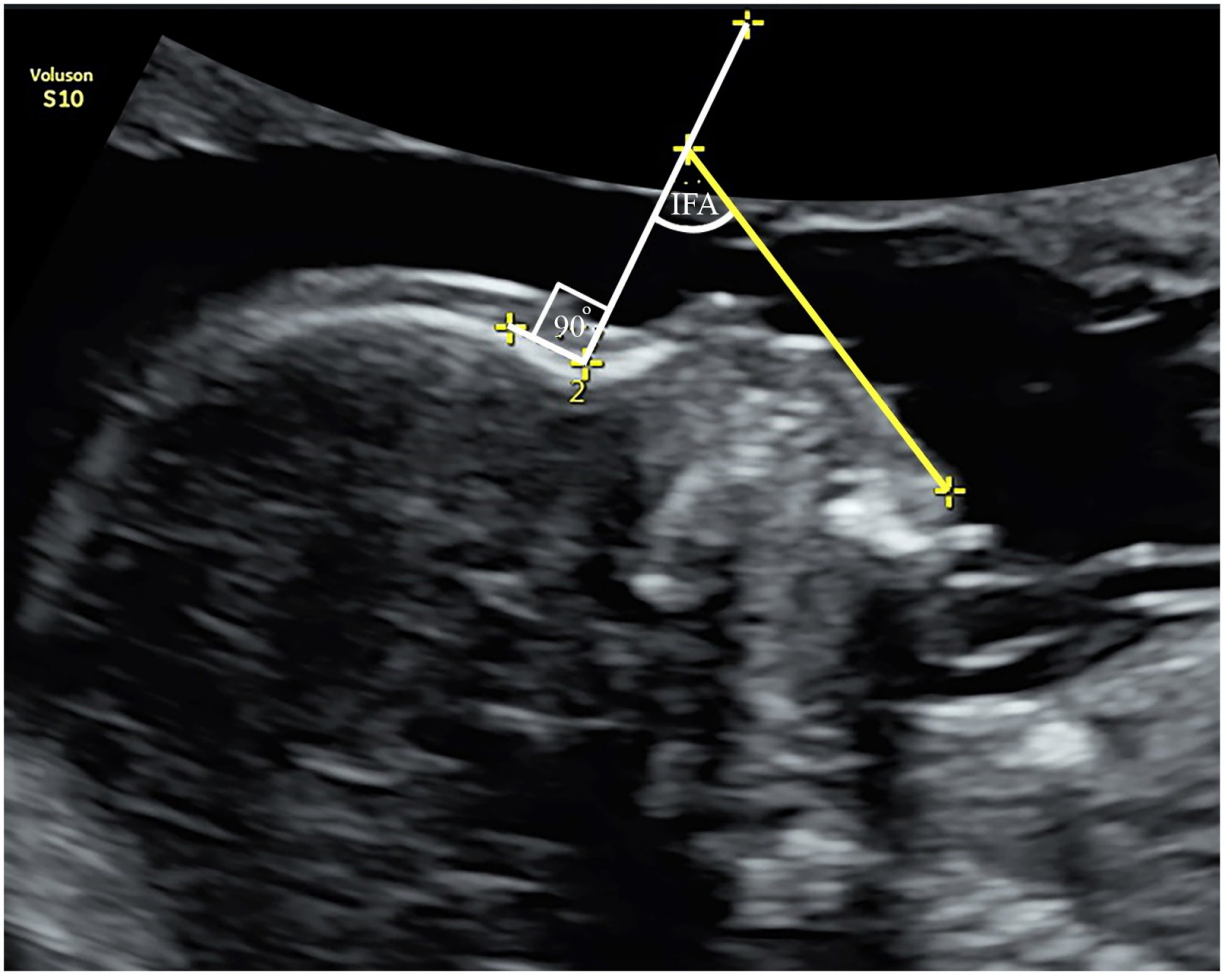
Inferior facial angle (IFA) measurement.

**Fig 2:**
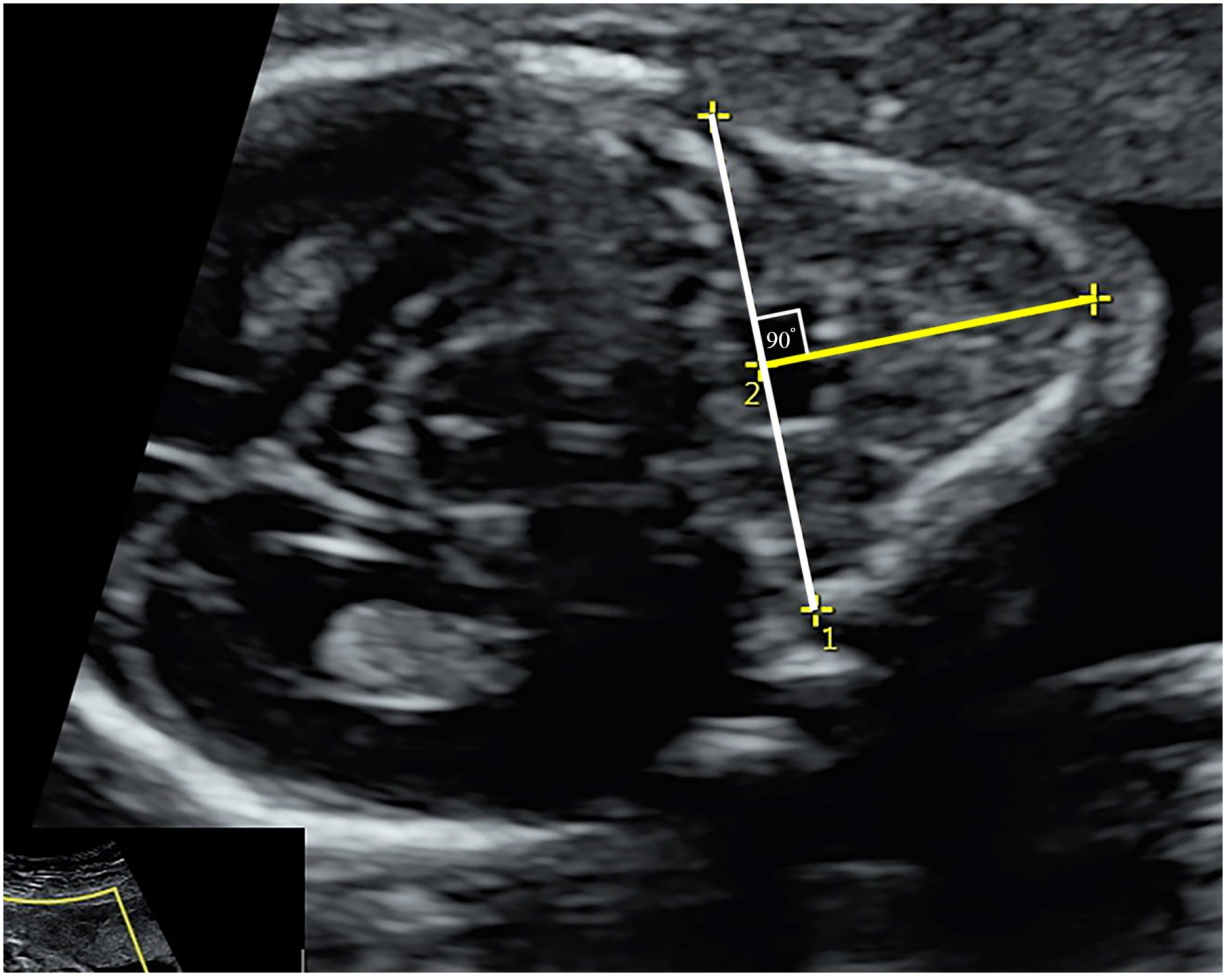
Anteroposterior mandibular diameter (APD) measurement. Jaw index = APD/biparietal diameter x 100.

**Fig 3:**
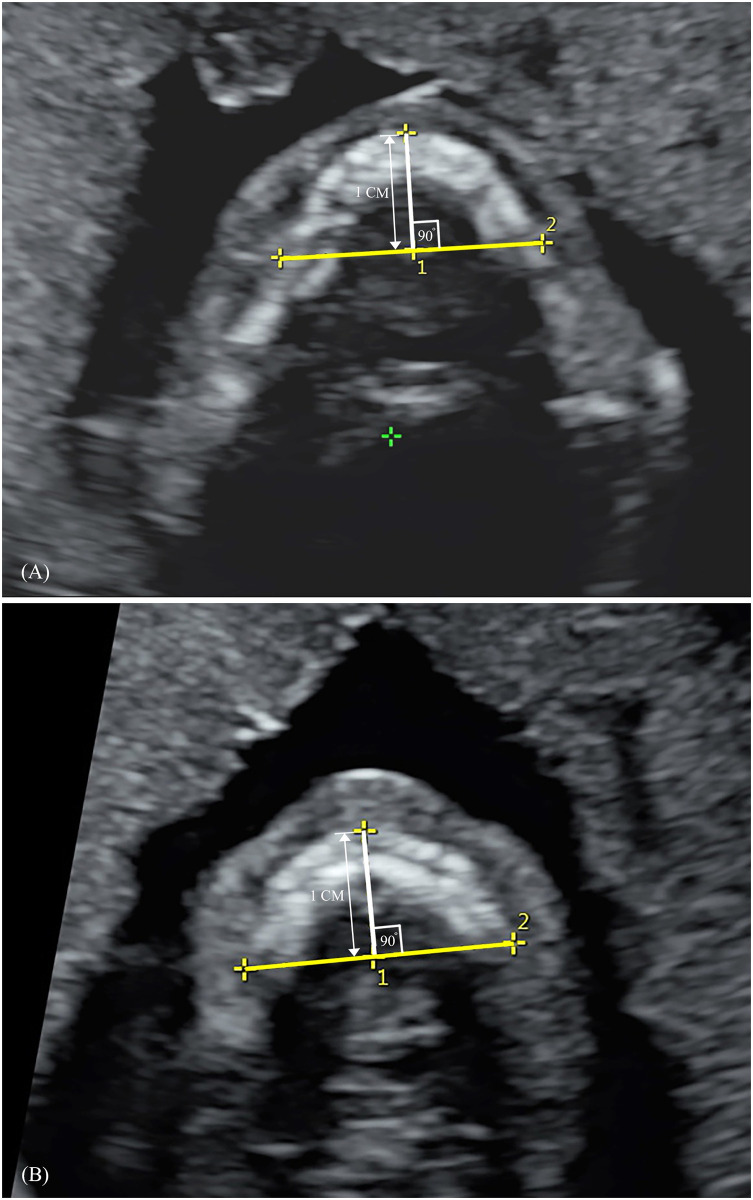
Mandible width and maxilla width measurements. (A) Mandible width (B) Maxilla width.

**Fig 4:**
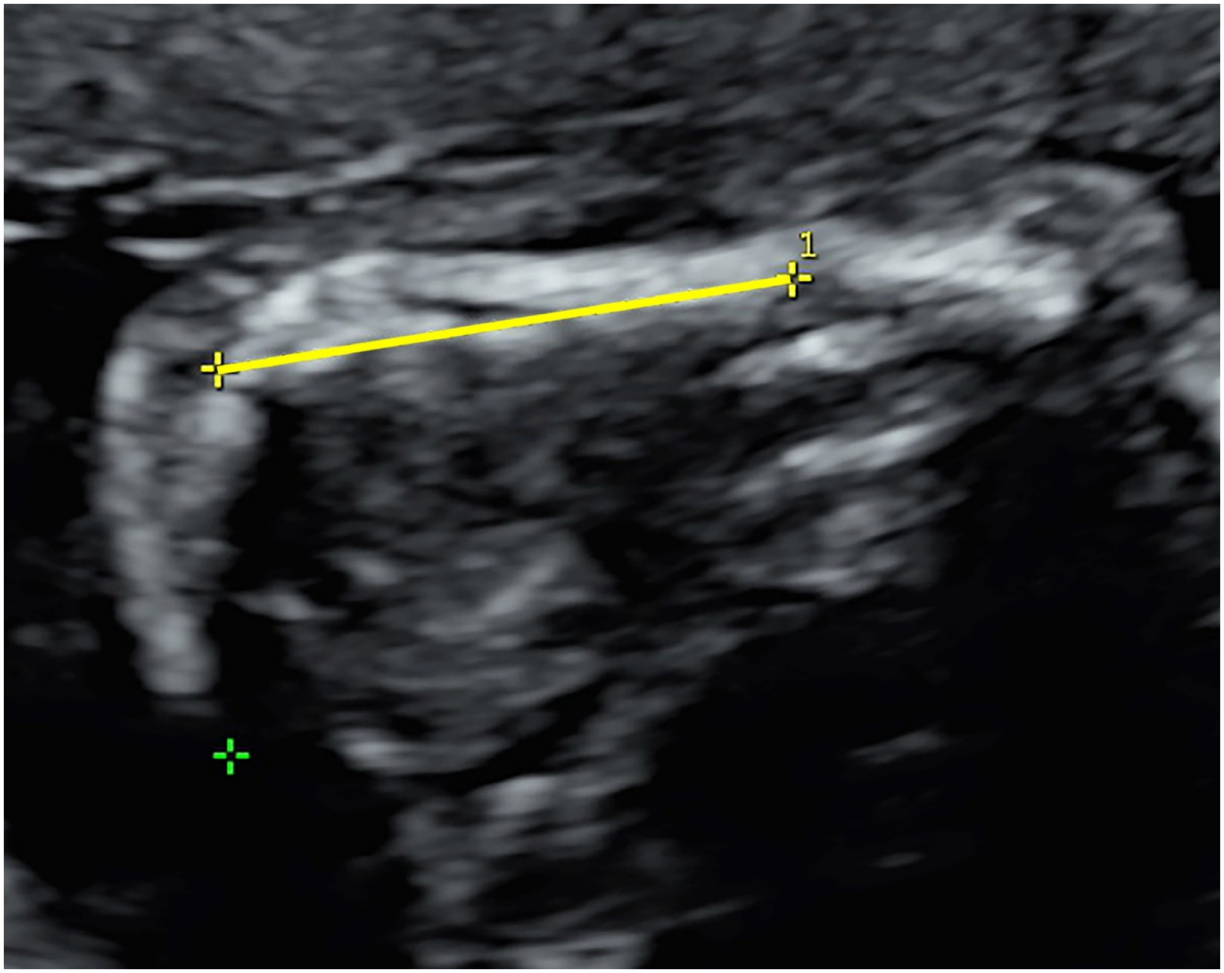
Mandible length (ML) measurements.

**Fig 5:**
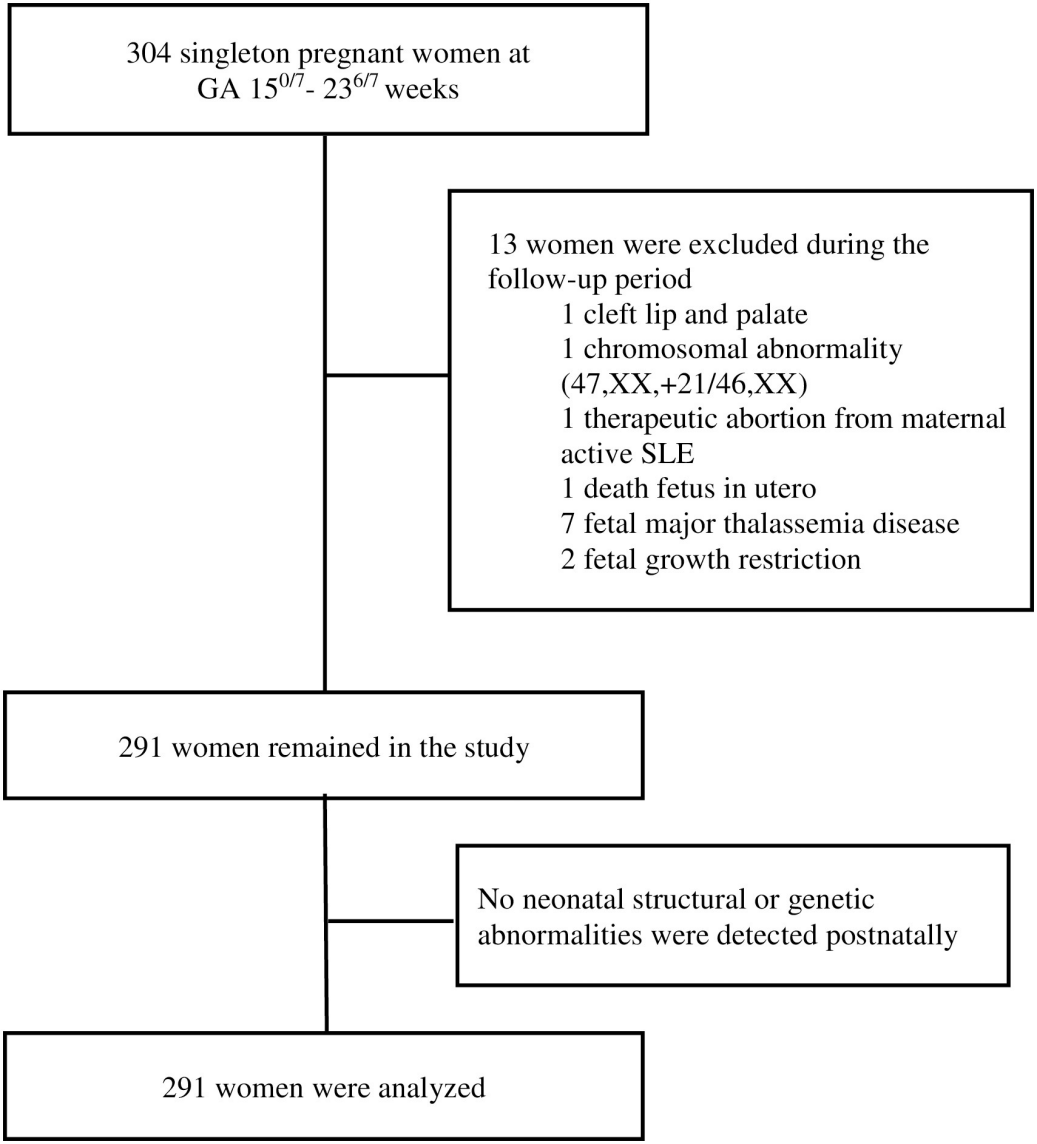
Flow diagram demonstrating woman enrollment.

The study was approved by the Ethics Committee of the Faculty of Medicine, Prince of Songkla University, Thailand (EC number 63-060-12-4) before its initiation, and was reviewed and approved by the Thai Clinical Trials Registry (TCTR) with identification number TCTR20210602003. The authors confirm that all related trials are also registered with the TCTR. This prospective study was conducted at the Maternal-Fetal Medicine Unit of Songklanagarind Hospital (a university hospital in Southern Thailand). The participants were recruited from April 1 to October 31, 2020. Singleton pregnant women at 15^0/7^ to 23^6/7^ weeks of gestation who attended the antenatal clinic or Maternal-Fetal Medicine Unit of Songklanagarind Hospital were invited to join the study. The exclusion criteria were 1) multifetal gestation confirmed by ultrasonography, 2) any suspicion of a congenital anomaly seen on the detailed ultrasonography at any gestational age, 3) genetic abnormality confirmed by any genetic testing, 4) fetal growth restriction following the consensus-based definition published in 2016 [20], 5) abnormal amniotic fluid volume (oligohydramnios defined as maximum vertical pocket < 2 cm or amniotic fluid index ≤ 5 cm, polyhydramnios defined as maximum vertical pocket ≥ 8 cm or amniotic fluid index ≥ 24 cm) [21], or 6) suspected fetal abnormality of the mandible, maxilla or jaws based on the proposed criteria from previous studies as follows: (1) IFA, evaluated in the mid-sagittal view of the fetal face, is defined as the angle at the intersection of a line drawn perpendicularly to the vertical part of the frontal bone at the level of the nasal bone synostosis and the line drawn from the tip of the mentum and the anterior border of the more protruding lip—An IFA < 49.2° is considered abnormal [13]; (2) jaw index, evaluated in the axial view, is the value calculated from the anteroposterior mandibular diameter (APD)/biparietal diameter x 100, in which the APD is the length between symphysis mentis and the laterolateral line joining bilateral bases of the mandible rami—A jaw index < 23 is considered abnormal [15]; (3) the MD/MX ratio, also evaluated in the axial view, is the diameter between the external surface of the mandible at the level 1 cm posteriorly from the anterior osseous border divided by the diameter measured in the same manner of the maxilla—An MD/MX ratio < 0.785 is considered abnormal [13]; and (4) ML, evaluated on the axial plane at a level slightly below the orbits, is the full length of mandible measured from the temporomandibular joint to the symphysis mentis—An ML < -2SD for gestational age is considered abnormal [19]. A woman was excluded immediately if any of the exclusion criteria were met, or omitted postnatally if the newborn was suspected of neonatal structural or genetic abnormalities which had been missed prenatally. All the women gave informed consents and were then categorized into 3 groups based on the gestational ages by ultrasonographic evaluations of biparietal diameter, head circumference, abdominal circumference and femur length at the time of mandible measurements, namely 15^0/7^–17^6/7^, 18^0/7^–20^6/7^ and 21^0/7^–23^6/7^ weeks of gestation. There was no discrepancy between the sonographic gestational ages assigned during the mandible measurements and the gestational ages assigned prior to the study based on our institution guideline, in which gestational ages were calculated from the last menstrual period and redated by ultrasonography. For gestational age ≤ 8^6/7^ weeks, 9^0/7^–15^6/7^ weeks, 16^0/7^–21^6/7^ weeks and 22^0/7^–23^6/7^ weeks calculated from the last menstrual period (LMP), the gestational age would be redated by ultrasonography if there were discrepancies between the LMP and ultrasound dating methods of more than 5 days, 7 days, 10 days and 14 days, respectively. All the women received ultrasonography to confirm gestational ages before 20 weeks of gestation. A Voluson S10 ultrasonographic machines (GE Healthcare, Chicago, IL), with a 2–5 MHz curvilinear transducer C1-5 -RS and also a Voluson E10 Digital Volume Ultrasound System, with 2–5 MHz broadband curved array probe C1-5D, were used during the study period. Transabdominal ultrasonographic measurements of fetal biometry, a detailed anatomical scan, and assessment of amniotic fluid volume were obtained for each patient. The target research ultrasound measurements consisted of 4 parameters, namely 1) inferior facial angle (IFA), 2) jaw index, 3) mandible width/ maxilla width ratio (MD/MX ratio), and 4) mandible length (ML). Our sonographic examination methods were based on the previous studies as mentioned in the exclusion criteria section [13, 15, 19], and are shown in Figs 1 to 5. Before conducting the study, we standardized all operators for fetal facial profile measurements to ensure acceptable intra-and-interobserver variabilities [22]. In this study, each woman was examined by one of the three operators (PB or RS or CS) during which three measurements were taken for each parameter within a limited scanning time of 30 minutes. The values of each parameter used in the analysis were the mean of the 3 measurements.

All women received standard antenatal care and were followed up until delivery. The last delivery was in February 2021. All newborns delivered at Songklanagarind Hospital were examined by neonatologists. Newborn examination records were retrieved from the Hospital Information System (HIS). The data of postnatal examinations of all newborns delivered in other hospitals were retrieved from the participants via telephone.

The sample size was calculated based on Leslie and Greenberg (1991) to be sure that the width of the precision of the 5th and 95th percentiles was not greater than one-fifth of the normal range [23]. Estimating that up to 10% of the women could be lost to follow-up, 330 patients were needed. The demographic and baseline patient characteristics are shown as number (%), mean (SD) or median (IQR) as appropriate. The distributions of each parameter are demonstrated by scatter plots against gestational age. Quantile regression was used to estimate changes in the median, 5th and 95th percentiles of each parameter across gestational age with the 95% confidence band around each percentile and to generate formulas for predicting the 5th percentile value for each parameter. The MD/MX ratio was log transformed before applying quantile regression analysis.

## Results

A total of 304 pregnant women were enrolled, 25 women had repeated measurements at different gestational ages, making a total of 330 measurements (24 women were measured twice, and 1 woman were measured three times). All the measurements values were initially included in the analysis and were categorized into 3 groups according to gestational age, 115, 106 and 109 women into 15^0/7^–17^6/7^, 18^0/7^–20^6/7^, 21^0/7^–23^6/7^ weeks groups, respectively. An initial check of the discrepancy between the results including all measurements and only those from analysis excluding subsequent measurements on the same woman revealed negligible differences in estimates of medians, 5^th^ and 95^th^ percentiles. However, to avoid the slight bias that might have been introduced from including all measurements, we reanalyzed using only the first time of measurement on each woman. There were 109, 97 and 98 women categorized into gestational age 15^0/7^–17^6/7^, 18^0/7^–20^6/7^, 21^0/7^–23^6/7^ weeks groups, respectively.

**Fig 6:**
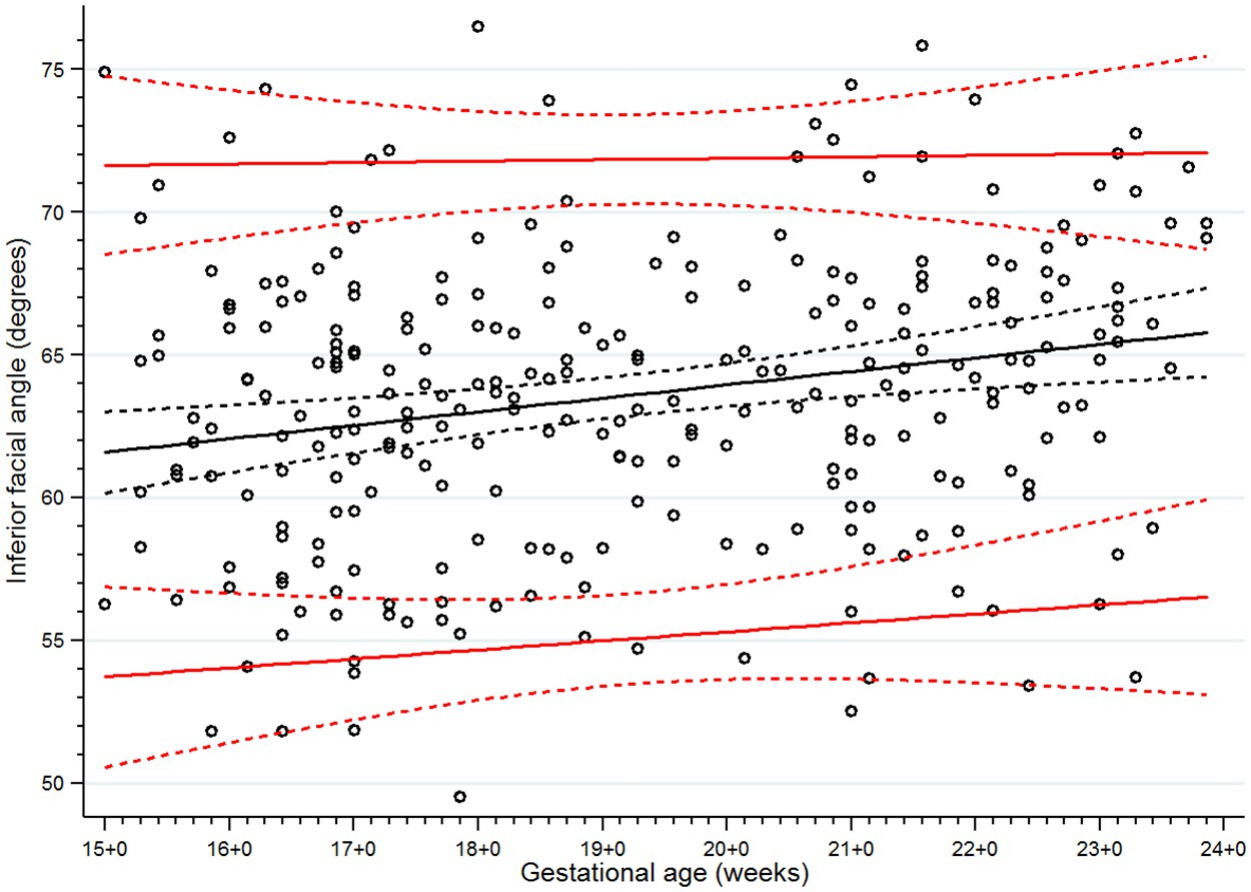
Scatter plot of inferior facial angle (IFA) along gestational age. Solid lines represent the IFA values in the 5th, 50th and 95th percentiles, Dashed lines represent the 95% confidence interval for each given percentile.

**Fig 7:**
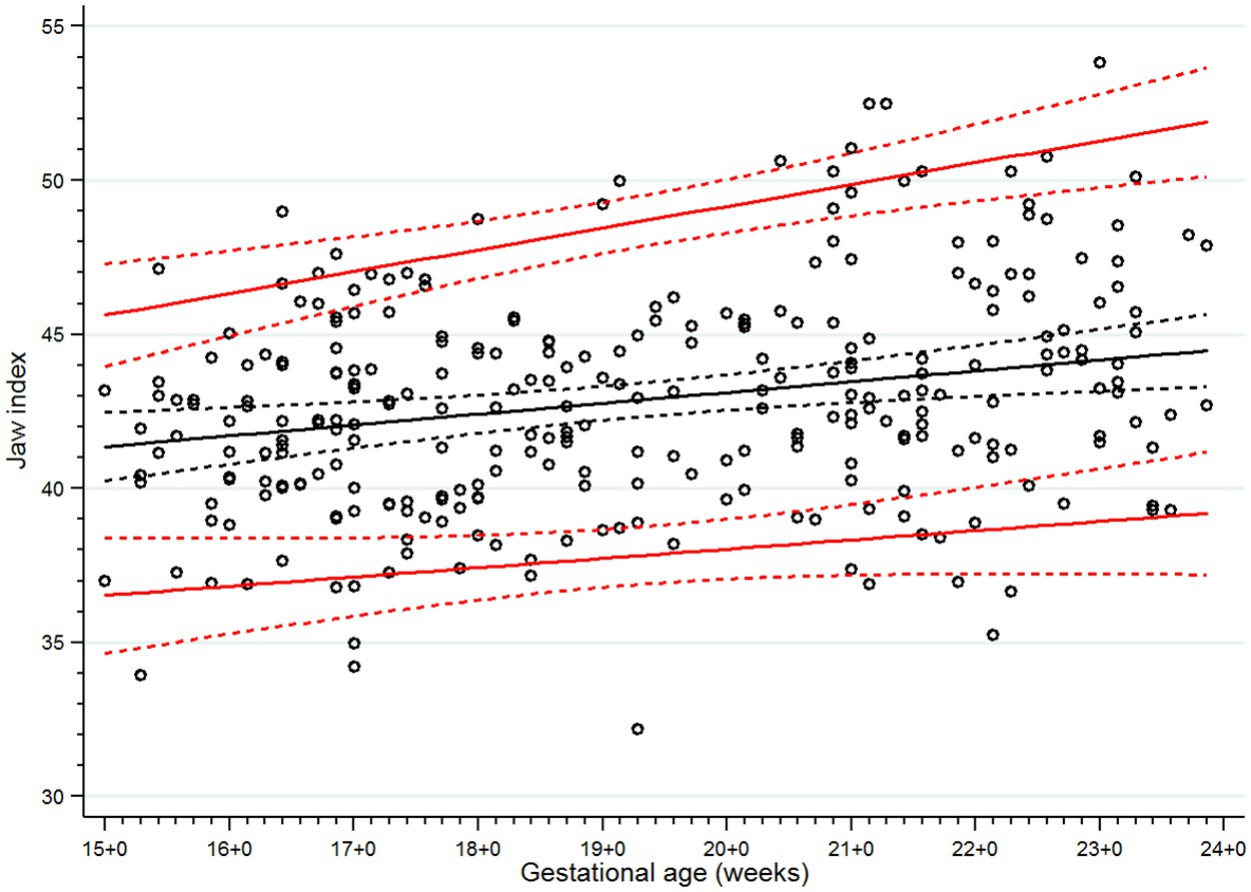
Scatter plot of jaw index along gestational age. Solid lines represent the jaw index values in the 5th, 50th and 95th percentiles, Dashed lines represent the 95% confidence interval for each given percentile.

**Fig 8:**
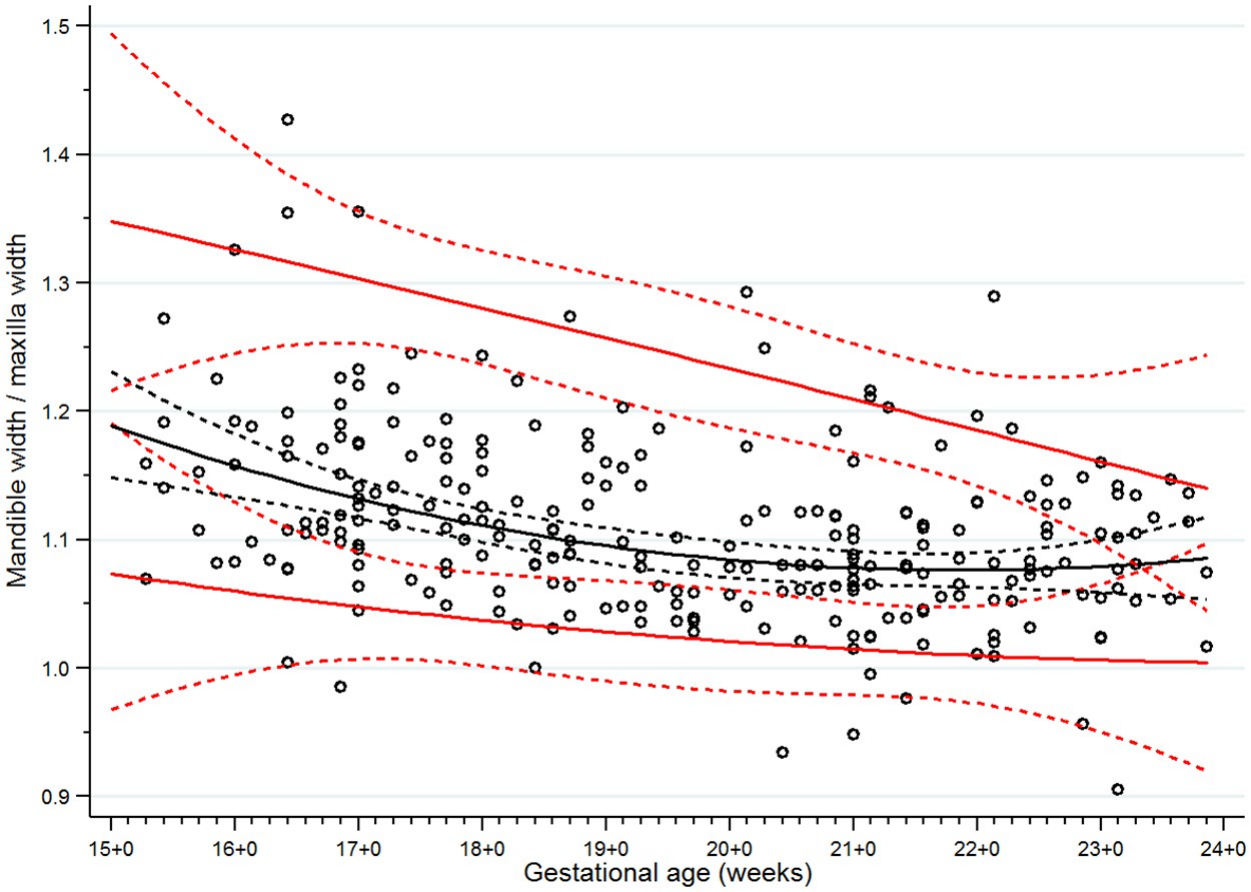
Scatter plot of mandible width/ maxilla width (MD/MX ratio) along gestational age. Solid lines represent the MD/MX ratio values in the 5th, 50th and 95th percentiles, Dashed lines represent the 95% confidence interval for each given percentile.

**Fig 9:**
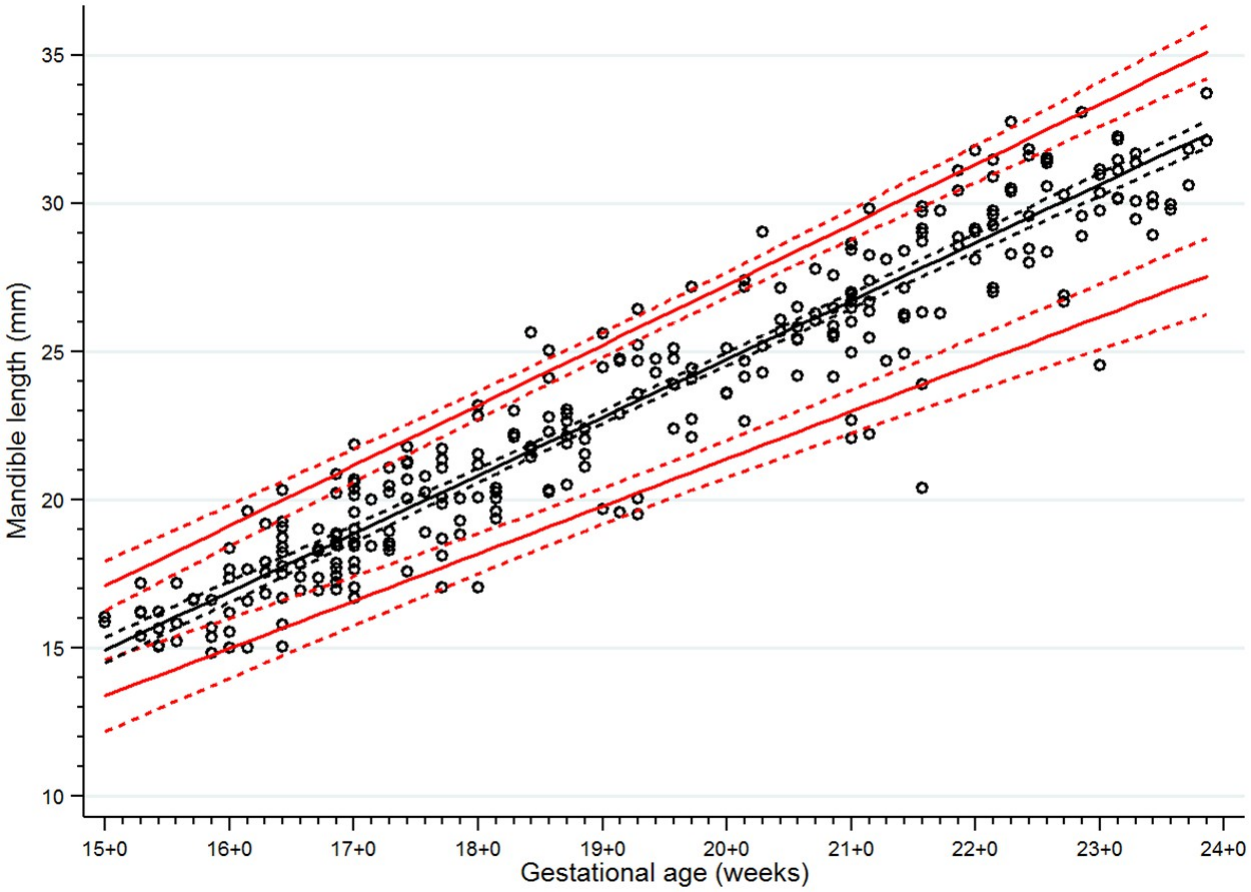
Scatter plot of mandible length (ML) along gestational age. Solid lines represent the ML values in the 5th, 50th and 95th percentiles, Dashed lines represent the 95% confidence interval for each given percentile.

There were 13 women excluded from the study for the following reasons: fetal cleft lip and palate seen on detailed ultrasonographic scan at 21 weeks of gestation (n = 1); chromosomal abnormality (47,XX,+21/46,XX) found from standard karyotyping of amniotic cells at 20 weeks (the indication for amniocentesis was fetal risk for major thalassemia disease, no anomalies were found from ultrasonography) (n = 1); therapeutic abortion due to maternal active systemic lupus erythematosus at 21 weeks of gestation (n = 1); dead fetus in utero at 26 weeks of gestation (n = 1); confirmed fetal major thalassemia disease, gestational age at exclusion at 18 to 22 weeks of gestation (n = 7); and fetal growth restriction diagnosed at 27 and 31 weeks of gestation (n = 2). None of the fetuses was suspected of a mandible abnormality using the criteria from the previously mentioned studies [13, 15, 18, 19]. We were able to follow all the remaining 291 participants until delivery (no loss to follow up). Most of the participants (238/291) delivered at our center (Songklanagarind Hospital), the rest were delivered at other hospitals. No women had home birth. None of the newborns was suspected of any structural or genetic abnormalities after physician’s examination. Finally, all 291 women who delivered structurally normal neonates were included in the analysis (Fig 5). The demographic data and obstetric outcomes are shown in Table 1. As there were some incomplete measurements of some parameters due to improper fetal position or thick maternal abdominal wall, the percentages of successful measurements of the IFA, jaw index, MD/MX ratio and ML were 93.8%, 98.3%, 87.3% and 99.7%, respectively. Scatter plots demonstrating the IFA, jaw index, MD/MX ratio and ML measurements across GA are shown in Figs 6 to 9, respectively. The IFA, jaw index and ML values significantly increased while the MD/MX ratio value significantly decreased with gestational age. The distributions at the 5th to 95th percentiles for IFA, jaw index, MD/MX ratio and ML for each gestational age are shown in Tables 2 to 5, respectively. Quantile regression was used to construct formulas predicting the 5th percentile value for each parameter. The equations (with 95% CI shown below) are as follows:

$$\text{IFA}=\underset{\left(53.62-56.62\right)}{55.12}+\underset{\left(-0.042-0.133\right)}{0.045}*(\text{GA}\;\text{in}\;\text{days}\;\text{-}\;136)$$

$$\text{jaw}\;\text{index}=\underset{\left(36.24-38.30\right)}{37.272}+\underset{\left(-0.044-0.078\right)}{0.01693}*(\text{GA}\;\text{in}\;\text{days}\;\text{-}\;136)$$

$$\text{MD/MX}\;\text{ratio}=\text{exp}\;\underset{\left(-0.0005-0.0538\right)}{(0.027}-\underset{\left(-0.00279-0.00059\right)}{0.00110}*(\text{GA}\;\text{in}\;\text{days}\;\text{-}\;136))$$

$$\text{ML}=\;\underset{\left(20.16-21.50\right)}{20.83}+\underset{\left(0.204-0.282\right)}{0.243}*(\text{GA}\;\text{in}\;\text{days}\;\text{-}\;136)$$

(Each parameter raw data is demonstrated separately in S1 Table).

**Table 1:** Demographic data and neonatal outcomes (N = 291).

	N (%), mean (SD) or median (IQR)
**Maternal characteristics**	
Age (years)	31.7 (5.2)
Gestational age at the time of performing ultrasonography (weeks)	
• 15^0/7^–17^6/7^	106 (36.4)
• 18^0/7^–20^6/7^	90 (30.9)
• 21^0/7^–23^6/7^	95 (32.6)
Ultrasonographic estimated fetal weight (g)	270 (187, 405)
**Neonatal outcomes**	
Gestational age at birth (weeks)	38.2 (1.6)
Preterm birth	35 (12)
Birthweight (g)	3083 (419.4)
Groups of birthweight	
• Normal	271 (93.1)
• Low birthweight (≤2500 g)	13 (4.5)
• Very low birthweight (≤1500 g)	7 (2.4)
APGAR score at 1 minutes	8.4 (0.8)
APGAR score at 5 minutes	8.9 (0.4)
APGAR score at 5 minutes < 7	1 (0.3)

**Table 2:** Reference range of inferior facial angle (IFA) as estimated by the quantile regression analysis with 95% confidence interval along gestational age (N = 273).

Gestational age (weeks)	5^th^ percentile, degrees (95% CI)	50^th^ percentile, degrees (95% CI)	95^th^ percentile, degrees (95% CI)
15 (n = 17)	53.9 (51.3–56.5)	62.1 (60.8–63.4)	71.7 (68.5–74.8)
16 (n = 44)	54.2 (52.0–56.3)	62.5 (61.4–63.6)	71.7 (69.1–74.3)
17 (n = 43)	54.5 (52.8–56.2)	62.9 (62.0–63.8)	71.8 (69.6–73.9)
18 (n = 38)	54.8 (53.3–56.3)	63.3 (62.6–64.0)	71.8 (70.0–73.6)
19 (n = 21)	55.1 (53.7–56.5)	63.7 (63.0–64.4)	71.9 (70.2–73.6)
20 (n = 21)	55.4 (53.9–57.0)	64.1 (63.3–64.9)	71.9 (70.0–73.8)
21 (n = 42)	55.8 (53.9–57.6)	64.5 (63.5–65.4)	72.0 (69.6–74.3)
22 (n = 24)	56.1 (53.7–58.4)	64.9 (63.7–66.1)	72.0 (69.2–74.9)
23 (n = 23)	56.4 (53.6–59.2)	65.3 (63.8–66.7)	72.1 (68.6–75.5)

**Table 3:** Reference range of jaw index as estimated by the quantile regression analysis with 95% confidence interval along gestational age (N = 286).

Gestational age (weeks)	5^th^ percentile (95% CI)	50^th^ percentile (95% CI)	95^th^ percentile (95% CI)
15 (n = 19)	36.7 (34.6–38.7)	41.7 (40.6–42.7)	45.9 (44.4–47.5)
16 (n = 45)	37.0 (35.3–38.7)	41.9 (41.0–42.8)	46.6 (45.4–47.9)
17 (n = 40)	37.3 (35.9–38.6)	42.2 (41.4–42.9)	47.3 (46.3–48.4)
18 (n = 39)	37.6 (36.4–38.7)	42.4 (41.8–43.0)	48.1 (47.2–48.9)
19 (n = 23)	37.9 (36.8–38.9)	42.6 (42.1–43.2)	48.8 (47.9–49.6)
20 (n = 25)	38.1 (36.9–39.4)	42.9 (42.3–43.5)	49.5 (48.5–50.4)
21 (n = 46)	38.4 (37.0–39.9)	43.1 (42.4–43.9)	50.2 (49.1–51.3)
22 (n = 25)	38.7 (36.9–40.5)	43.4 (42.4–44.3)	50.9 (49.5–52.2)
23 (n = 24)	39.0 (36.8–41.2)	43.6 (42.5–44.8)	51.6 (49.9–53.2)

**Table 4:** Reference range of mandible width/maxilla width ratio (MD/MX ratio) as estimated by back transformed from quantile regression analysis of ln(MD/MX ratio) with 95% confidence interval along gestational age (N = 254).

Gestational age (weeks)	5^th^ percentile (95% CI)	50^th^ percentile (95% CI)	95^th^ percentile (95% CI)
15 (n = 8)	1.06 (1.00–1.12)	1.14 (1.12–1.16)	1.30 (1.24–1.37)
16 (n = 30)	1.05 (1.00–1.10)	1.13 (1.11–1.15)	1.28 (1.23–1.34)
17 (n = 36)	1.04 (1.00–1.08)	1.12 (1.11–1.13)	1.27 (1.23–1.31)
18 (n = 42)	1.03 (1.00–1.07)	1.11 (1.10–1.12)	1.25 (1.22–1.28)
19 (n = 24)	1.03 (1.00–1.06)	1.10 (1.09–1.11)	1.23 (1.20–1.26)
20 (n = 25)	1.02 (0.99–1.05)	1.09 (1.08–1.10)	1.22 (1.19–1.25)
21 (n = 45)	1.01 (0.98–1.05)	1.08 (1.07–1.09)	1.20 (1.16–1.24)
22 (n = 21)	1.01 (0.96–1.05)	1.07 (1.05–1.08)	1.18 (1.14–1.23)
23 (n = 23)	1.00 (0.95–1.06)	1.06 (1.04–1.08)	1.17 (1.12–1.22)

**Table 5:** Reference range of mandible length (ML) as estimated by the quantile regression analysis with 95% confidence interval along gestational age (N = 290).

Gestational age (weeks)	5^th^ percentile (mm) (95% CI)	50^th^ percentile (mm) (95% CI)	95^th^ percentile (mm) (95% CI)
15 (n = 19)	14.1 (12.9–15.2)	15.8 (15.3–16.2)	18.0 (17.2–18.7)
16 (n = 45)	15.7 (14.7–16.6)	17.7 (17.4–18.1)	20.0 (19.4–20.6)
17 (n = 43)	17.3 (16.5–18.0)	19.7 (19.4–20.0)	22.0 (21.5–22.5)
18 (n = 36)	18.9 (18.2–19.5)	21.7 (21.4–21.9)	24.1 (23.7–24.5)
19 (n = 25)	20.5 (19.9–21.1)	23.7 (23.4–23.9)	26.1 (25.7–26.5)
20 (n = 26)	22.1 (21.4–22.7)	25.6 (25.4–25.9)	28.1 (27.7–28.6)
21 (n = 42)	23.7 (22.9–24.5)	27.6 (27.3–27.9)	30.2 (29.6–30.7)
22 (n = 28)	25.3 (24.3–26.3)	29.6 (29.2–29.9)	32.2 (31.5–32.8)
23 (n = 26)	26.9 (25.7–28.1)	31.5 (31.1–32.0)	34.2 (33.4–35.0)

## Discussion

We developed the reference ranges for fetal mandibular measurements of normal Thai fetuses and created the formulas to calculate the 5th percentile values of these mandible parameters to use as the lower normal limits. We found that IFA, jaw index and ML significantly increased across gestational age, while the MD/MX ratio significantly decreased.

The reference ranges we established are somewhat similar to the reported values from previous studies in France, China, Italy, England, German and the USA from 1993–2018 [13–15, 17–19], however there were some differences. A study in Chinese women by Lu et al. (2019) reported similar IFA values as in this study, especially at 16 to 18 weeks of gestational age. The same study also found that the IFA values increased with advancing gestational age but in more obvious way, at gestational age 19 to 23 weeks our IFA values were lower [14]. Our overall IFA values were more similar to the study by Rotten et al. (2002) conducted in France, but they found that the IFA values were constant, while we found increasing values across gestational age. The MD/MX ratio values reported in the same study were also similar to our study but they were constant across gestational age 18 to 28 weeks, contrary to the decreasing values in this study [13]. Studies by Chitty et al. (1993) [17], Neuschulz et al. (2015) [18] and Otto and Platt (1991) [19], conducted in England, Germany and the USA, respectively, reported similar ML values to our study, while a study by Lai and Yeo (1995) in Singapore reported slightly lower ML values [16]. All studies found that ML values increased along gestational age [16–19]. Since the measurement methods of each parameter was the same, we believe that the differences regarding the differences values along advancing gestational age are probably related to ethnic facial determinants.

Among the 13 women who had been excluded, there were a case of fetal cleft lip and palate and a case with chromosomal abnormality (47,XX,+21/46,XX). Unfortunately, we were unable to obtain the IFA values from the fetus with cleft lip and palate due to improper fetal position during the measurement at 17 weeks of gestation. The diagnosis of cleft lip and palate was made afterward at 21 weeks of gestation during the repeated detailed anatomical scan as a standard of care. As reported by Rotten et al. (2002), the IFA values in cleft lip/palate fetuses were in the normal range, but there could be subjective impression of retrognathia in these fetuses due to protruding cutaneous profile [13]. For the fetus with 47,XX,+21/46,XX, and also other excluded fetuses due to dead fetus in utero, FGR, major thalassemia diseases and maternal SLE, all the parameters were within our reported reference ranges.

The strength of our study was that it was prospective, so that there was no information bias from inadequate medical records regarding either antenatal or postnatal physical examinations of the neonates. To our knowledge, this is the first study on the reference ranges of the fetal mandible parameters in Thailand. We evaluated four proposed parameters to determine normal mandible development both in size and position. At the beginning of this study, the values of each parameter from each sonographer were evaluated and resulted in moderate-to-excellent intra- and-inter-observer agreements [22]. The major limitation of our study was the incomplete measurement of some parameters due to poor fetal position or thick maternal abdominal wall.

Even though the values of these parameters have not been studied with micrognathia fetuses, it would be appropriate to suspect mandible abnormalities and perform a thorough fetal anatomical scan in women whose fetuses are found to have measurement values less than the 5th percentile to ensure the normal process of facial development. Further research to validate these measurement values in fetuses with abnormal mandibles would bring greater understanding about the validity of these measurements for use in a clinical setting.

## Conclusion

The reference ranges and formulas to calculate the 5th percentile values of mandible parameters were developed. The use of these lower normal limit values can provide a stronger basis for diagnosing fetal micro- and retrognathia than simple subjective examination. We found that IFA, jaw index and ML increased significantly along gestational age, while the MD/MX ratio significantly decreased.

## Supporting information

S1 Table Demonstrating measurement values of inferior facial angle (IFA), jaw index, mandible width/maxilla width ratio (MD/MX ratio), and mandible length (ML) (N = 291). (DOCX)
