# Supplementary material for: Reference ranges of fetal mandible measurements: Inferior facial angle, jaw index, mandible width/maxilla width ratio and mandible length in Thai fetuses at 15 to 23 weeks of gestation
Source: PLoS One. 2022 Jun 1;17(6):e0269095. doi: 10.1371/journal.pone.0269095 (PMC9159587; doi:10.1371/journal.pone.0269095)
Supplement: S1 Table — (DOCX) [file pone.0269095.s001.docx]

**S1 Table demonstrating measurement values of inferior facial angle (IFA), jaw index, mandible width/maxilla width ratio (MD/MX ratio), and mandible length (ML) (N = 291)**

| **ID** | **Gestational age by ultrasound (weeks)** | **Estimated fetal weight (g)** | **IFA (degrees)** | **Jaw index** | **Mandible width (mm)** | **Maxilla width (mm)** | **MD/MX ratio** | **ML (mm)** |
| --- | --- | --- | --- | --- | --- | --- | --- | --- |
| **1** | 18 | 203 | 58.53 | 39.67 | 21.37 | 19.17 | 1.11 | 21.20 |
| **2** | 23 | 514 | 70.92 | 41.70 | 24.57 | 23.30 | 1.05 | 30.97 |
| **3** | 17 | 206 | NA | 43.83 | 18.05 | 13.32 | 1.36 | 20.35 |
| **4** | 19.3 | 306 | NA | 45.89 | 19.97 | 18.77 | 1.06 | 24.77 |
| **5** | 18 | 201 | 63.95 | 38.47 | 20.00 | 17.13 | 1.17 | 17.07 |
| **6** | 21 | 433 | 74.45 | 42.12 | 21.63 | 21.10 | 1.03 | 26.67 |
| **7** | 15.6 | 108 | 51.82 | 39.49 | 16.23 | NA | NA | 14.83 |
| **8** | 21.3 | 391 | 57.95 | 41.68 | 23.73 | 21.17 | 1.12 | 26.27 |
| **9** | 20.1 | 320 | 54.37 | 39.93 | 24.78 | 19.17 | 1.29 | 22.67 |
| **10** | 23.5 | 625 | NA | NA | 26.48 | 23.32 | 1.14 | 30.60 |
| **11** | 22.2 | 507 | 68.11 | 46.95 | 25.10 | 23.50 | 1.07 | 32.77 |
| **12** | 18.1 | 223 | 64.04 | 38.14 | 19.67 | 18.57 | 1.06 | 20.03 |
| **13** | 19.5 | 299 | 68.07 | 40.46 | 20.60 | 20.03 | 1.03 | 22.10 |
| **14** | 18.1 | 196 | 63.67 | 42.61 | 20.83 | 18.90 | 1.10 | 19.63 |
| **15** | 21 | 398 | 58.84 | 40.80 | 22.80 | 21.50 | 1.06 | 28.63 |
| **16** | 23 | 462 | 65.71 | 41.50 | 22.67 | 22.13 | 1.02 | 24.55 |
| **17** | 18 | 219 | 66.00 | 44.53 | 20.30 | 18.03 | 1.13 | 21.53 |
| **18** | 18.4 | 252 | 58.18 | 44.78 | 20.93 | 19.63 | 1.07 | 24.10 |
| **19** | 16.3 | 123 | 55.17 | 41.56 | 17.48 | 12.25 | 1.43 | 15.07 |
| **20** | 21.4 | 383 | 65.16 | 44.22 | 23.40 | 21.10 | 1.11 | 29.13 |
| **21** | 17.3 | 200 | 62.46 | 37.89 | 22.53 | NA | NA | 21.23 |
| **22** | 23 | 494 | 56.25 | 46.03 | 24.93 | 21.50 | 1.16 | 29.77 |
| **23** | 16.3 | 170 | 60.93 | 40.09 | 17.77 | 16.50 | 1.08 | 19.07 |
| **24** | 16.3 | 134 | 62.16 | 37.63 | 19.55 | 14.43 | 1.35 | 15.80 |
| **25** | 20.4 | 354 | 71.92 | 39.04 | 20.35 | 18.83 | 1.08 | 26.52 |
| **26** | 23.6 | 600 | 69.60 | 47.89 | 24.00 | 22.33 | 1.07 | 32.10 |
| **27** | 22.4 | 508 | 67.88 | 44.93 | 27.17 | 23.70 | 1.15 | 30.57 |
| **28** | 23.4 | 460 | 69.60 | 39.31 | 25.50 | 22.23 | 1.15 | 29.80 |
| **29** | 19.4 | 296 | 59.36 | 38.18 | 23.73 | 22.40 | 1.06 | 24.77 |
| **30** | 20.2 | 430 | 64.40 | 42.60 | 23.40 | 18.73 | 1.25 | 29.03 |
| **31** | 16.6 | 157 | 59.47 | 43.75 | 17.95 | NA | NA | 17.70 |
| **32** | 17 | 223 | 67.37 | 40.01 | 20.40 | 18.88 | 1.08 | 20.15 |
| **33** | 22.6 | 636 | 63.22 | 44.16 | 23.47 | 22.20 | 1.06 | 33.07 |
| **34** | 22.3 | 538 | 60.06 | 46.24 | 24.63 | 22.73 | 1.08 | 29.57 |
| **35** | 19.2 | 272 | 64.83 | 32.18 | 21.37 | 20.63 | 1.04 | 19.50 |
| **36** | 17 | 196 | 53.84 | NA | 19.37 | 17.37 | 1.12 | 21.87 |
| **37** | 20.6 | 342 | 60.47 | 43.77 | 22.23 | 19.87 | 1.12 | 25.87 |
| **38** | 20.5 | 373 | 66.47 | 38.97 | 22.87 | 21.57 | 1.06 | 26.03 |
| **39** | 17 | 169 | 67.08 | 34.95 | 19.03 | 17.90 | 1.06 | 18.45 |
| **40** | 19.2 | 298 | 61.25 | 38.89 | 19.47 | 16.70 | 1.17 | 20.03 |
| **41** | 17 | 188 | 62.38 | 42.06 | 18.72 | 17.08 | 1.10 | 18.72 |
| **42** | 20.3 | 344 | NA | 43.59 | 21.42 | 22.93 | 0.93 | 26.07 |
| **43** | 19.5 | 326 | 62.17 | 45.27 | 20.03 | 19.33 | 1.04 | 22.73 |
| **44** | 18.6 | 269 | 56.84 | 40.52 | 19.27 | 16.43 | 1.17 | 21.10 |
| **45** | 22 | 377 | NA | 46.65 | 22.10 | 19.57 | 1.13 | 29.13 |
| **46** | 22.4 | 472 | 65.28 | 48.74 | 24.33 | 22.63 | 1.08 | 31.37 |
| **47** | 16.6 | 182 | 62.27 | 36.79 | 19.18 | 15.65 | 1.23 | 17.22 |
| **48** | 19 | 218 | 62.21 | 38.65 | 20.80 | 18.22 | 1.14 | 19.68 |
| **49** | 22 | 513 | 73.92 | 41.62 | 23.90 | 21.17 | 1.13 | 31.80 |
| **50** | 18.4 | 234 | 66.83 | 43.47 | 20.03 | 19.43 | 1.03 | 20.33 |
| **51** | 18.5 | 275 | 68.79 | 41.65 | 19.33 | 18.18 | 1.06 | 22.92 |
| **52** | 21.1 | 407 | 64.71 | 52.49 | 23.57 | 21.83 | 1.08 | 29.83 |
| **53** | 21 | 361 | NA | 44.06 | 23.10 | 22.77 | 1.01 | 24.97 |
| **54** | 20.6 | 346 | 66.88 | 49.09 | 21.80 | 20.50 | 1.06 | 27.57 |
| **55** | 18.6 | 318 | 55.12 | 44.28 | 22.30 | 18.87 | 1.18 | 22.05 |
| **56** | 17.2 | 178 | 63.65 | 42.73 | 20.12 | 17.63 | 1.14 | 21.07 |
| **57** | 22.5 | 486 | 63.17 | 44.40 | NA | 21.57 | NA | 26.90 |
| **58** | 18 | 220 | 76.51 | 44.39 | 21.47 | 18.23 | 1.18 | 23.20 |
| **59** | 17 | 171 | 51.85 | 39.25 | 18.28 | 17.50 | 1.04 | 18.53 |
| **60** | 16.3 | 162 | 51.80 | 41.40 | NA | NA | NA | 18.23 |
| **61** | 16.6 | 169 | 64.71 | 43.72 | 16.95 | 15.33 | 1.11 | 18.85 |
| **62** | 21.4 | 310 | 67.74 | 42.48 | 21.47 | 20.00 | 1.07 | 23.90 |
| **63** | 23.1 | 736 | 57.98 | 43.46 | 27.40 | 24.13 | 1.14 | 32.13 |
| **64** | 21.6 | 466 | 58.83 | 41.21 | 23.43 | 21.17 | 1.11 | 28.57 |
| **65** | 18.6 | 264 | 65.92 | 42.02 | 21.27 | 18.53 | 1.15 | 22.40 |
| **66** | 22.2 | 447 | 64.81 | 41.27 | 24.03 | NA | NA | 28.30 |
| **67** | 20.6 | 368 | NA | 48.00 | 23.43 | 21.23 | 1.10 | 24.13 |
| **68** | 21.5 | 425 | 62.79 | 43.04 | 24.87 | 21.20 | 1.17 | 29.77 |
| **69** | 16.6 | 188 | 64.57 | 41.89 | 17.35 | NA | NA | 18.48 |
| **70** | 17.5 | 187 | 60.43 | 38.90 | 20.17 | 17.17 | 1.17 | 21.38 |
| **71** | 18.4 | 204 | 68.05 | 41.63 | 19.30 | 17.20 | 1.12 | 20.25 |
| **72** | 18.1 | 201 | 60.24 | 40.55 | 19.45 | NA | NA | 20.27 |
| **73** | 16.6 | 146 | 65.06 | 44.54 | 18.18 | 16.25 | 1.12 | 17.87 |
| **74** | 18.3 | 214 | 69.57 | 43.52 | 19.70 | 16.57 | 1.19 | 21.70 |
| **75** | 21 | 317 | 63.37 | 43.88 | 23.30 | 21.40 | 1.09 | 26.00 |
| **76** | 17.3 | 189 | 61.55 | 43.08 | 17.75 | NA | NA | 20.03 |
| **77** | 17.4 | 217 | 65.20 | 39.06 | 20.28 | 17.23 | 1.18 | 20.78 |
| **78** | 23.1 | 585 | 67.35 | 44.03 | 20.97 | 23.17 | 0.91 | 32.27 |
| **79** | 18.4 | 202 | 62.32 | 44.42 | 18.90 | 17.40 | 1.09 | 25.03 |
| **80** | 22.1 | 589 | 66.84 | 41.43 | 25.40 | 25.17 | 1.01 | 31.47 |
| **81** | 21.1 | 366 | 58.19 | 36.90 | 23.25 | 19.12 | 1.22 | 27.38 |
| **82** | 22.3 | 458 | 64.80 | 40.07 | 22.80 | 22.10 | 1.03 | 28.47 |
| **83** | 18.5 | 276 | 62.70 | 42.66 | 18.93 | 17.40 | 1.09 | 21.90 |
| **84** | 16.6 | 221 | 65.86 | 45.40 | 17.67 | 17.93 | 0.99 | 20.88 |
| **85** | 19.2 | 365 | 63.08 | 41.20 | 20.42 | 19.48 | 1.05 | 25.22 |
| **86** | 16.5 | 148 | 57.74 | 42.12 | 18.27 | 15.60 | 1.17 | 18.33 |
| **87** | 18.3 | 215 | NA | 37.68 | 19.10 | 17.43 | 1.10 | 21.80 |
| **88** | 22.1 | 460 | 56.03 | 42.79 | 23.37 | 22.20 | 1.05 | 29.60 |
| **89** | 20.1 | 345 | 65.12 | 45.22 | 22.77 | 21.13 | 1.08 | 27.40 |
| **90** | 21.3 | 401 | 62.16 | 41.60 | 23.03 | 21.37 | 1.08 | 28.40 |
| **91** | 18.2 | 206 | 63.48 | 45.54 | 20.63 | 18.27 | 1.13 | 22.23 |
| **92** | 21.6 | 481 | 60.50 | 46.99 | 24.37 | 23.07 | 1.06 | 31.10 |
| **93** | 16 | 117 | 72.62 | 41.19 | 16.20 | 13.58 | 1.19 | 15.00 |
| **94** | 20.5 | 372 | 63.64 | 47.32 | 23.33 | 20.80 | 1.12 | 26.30 |
| **95** | 19 | 315 | 58.22 | 43.57 | 22.27 | 19.20 | 1.16 | 25.60 |
| **96** | 18.5 | 244 | 70.39 | 41.49 | 19.57 | 17.97 | 1.09 | 23.03 |
| **97** | 21.4 | 460 | 68.27 | 42.09 | 22.20 | 20.27 | 1.10 | 29.70 |
| **98** | 15.2 | 113 | 64.78 | 41.92 | 16.97 | 14.63 | 1.16 | 16.22 |
| **99** | 22.1 | 435 | 67.17 | 41.00 | 25.03 | 24.53 | 1.02 | 29.77 |
| **100** | 22.1 | 462 | 70.77 | 35.25 | 23.73 | 23.13 | 1.03 | 27.13 |
| **101** | 17.5 | 189 | 63.55 | 44.92 | 18.65 | 17.25 | 1.08 | 21.07 |
| **102** | 15.2 | 151 | 58.28 | 40.17 | NA | NA | NA | 17.20 |
| **103** | 17.1 | 210 | 60.19 | 43.85 | 19.32 | 17.00 | 1.14 | 20.00 |
| **104** | 20.3 | 355 | 69.19 | 45.76 | 22.10 | 20.47 | 1.08 | 27.13 |
| **105** | 23.5 | 557 | 71.57 | 48.23 | 24.70 | 22.17 | 1.11 | 31.83 |
| **106** | 19.1 | 265 | 62.65 | 43.39 | 20.13 | 18.33 | 1.10 | 24.67 |
| **107** | 22.3 | 487 | 53.40 | 48.86 | 24.20 | 22.47 | 1.08 | 31.60 |
| **108** | 15.3 | 135 | 70.94 | 41.15 | 19.08 | 15.00 | 1.27 | 15.03 |
| **109** | 19.4 | 331 | 69.14 | 43.13 | 21.80 | 20.77 | 1.05 | 25.10 |
| **110** | 16.5 | 164 | 61.80 | 46.98 | 19.20 | 17.33 | 1.11 | 19.00 |
| **111** | 20 | 323 | 64.80 | 39.63 | 21.63 | 20.47 | 1.06 | 23.57 |
| **112** | 15.4 | 116 | 60.77 | 42.85 | NA | NA | NA | 15.83 |
| **113** | 17.5 | 202 | 62.50 | 39.62 | 20.02 | 18.05 | 1.11 | 19.87 |
| **114** | 18.1 | 200 | 56.19 | 41.22 | 19.97 | 17.97 | 1.11 | 20.40 |
| **115** | 17.5 | 218 | 57.52 | 41.33 | 19.32 | 17.98 | 1.07 | 18.13 |
| **116** | 22.1 | 479 | 63.30 | 45.77 | 23.83 | 23.37 | 1.02 | 30.90 |
| **117** | 21 | 374 | 65.99 | 42.40 | 19.43 | 20.50 | 0.95 | 26.47 |
| **118** | 23.3 | 553 | 66.08 | 41.33 | NA | NA | NA | 29.97 |
| **119** | 17.3 | 180 | 65.88 | 39.58 | 19.92 | 17.10 | 1.16 | 17.57 |
| **120** | 15.4 | 118 | 60.95 | 41.69 | 14.87 | NA | NA | 15.23 |
| **121** | 16.4 | 153 | 62.87 | 40.11 | 18.33 | NA | NA | 17.42 |
| **122** | 16.3 | 221 | NA | 40.02 | 18.85 | 16.02 | 1.18 | 19.25 |
| **123** | 19.5 | 323 | 67.00 | 44.73 | 21.57 | 20.77 | 1.04 | 24.43 |
| **124** | 16.6 | 155 | 55.90 | 39.03 | 17.48 | 14.50 | 1.21 | 17.43 |
| **125** | 16.6 | 116 | 68.55 | 47.60 | NA | NA | NA | 16.97 |
| **126** | 17 | 152 | 61.34 | 41.57 | 19.42 | 15.92 | 1.22 | 17.03 |
| **127** | 17 | 152 | 69.45 | 34.20 | 18.18 | 16.07 | 1.13 | 17.90 |
| **128** | 17.2 | 211 | 61.74 | 39.45 | 18.40 | NA | NA | 20.25 |
| **129** | 16.5 | 157 | 64.72 | 40.45 | 16.07 | NA | NA | 16.93 |
| **130** | 22.5 | 513 | 67.58 | 39.48 | 24.80 | 22.93 | 1.08 | 26.67 |
| **131** | 17.2 | 202 | 72.15 | 42.84 | 19.90 | 16.70 | 1.19 | 18.45 |
| **132** | 19.3 | 341 | 68.21 | 45.44 | 21.60 | 18.20 | 1.19 | 24.30 |
| **133** | 17.5 | 211 | 56.33 | 43.73 | 18.63 | 16.27 | 1.15 | 18.70 |
| **134** | 16 | 143 | 66.62 | 45.03 | 16.57 | 15.30 | 1.08 | 18.37 |
| **135** | 21.6 | 596 | 64.65 | 36.97 | 26.07 | 24.47 | 1.07 | 28.87 |
| **136** | 16.6 | 174 | 60.72 | 45.53 | 18.40 | 16.75 | 1.10 | 18.58 |
| **137** | 16.3 | 202 | 58.64 | 46.64 | 18.98 | 18.90 | 1.00 | 20.33 |
| **138** | 15.6 | 127 | 60.75 | 44.25 | 18.97 | 15.48 | 1.22 | 16.62 |
| **139** | 22.1 | 439 | 68.30 | 46.42 | 24.20 | 18.77 | 1.29 | 29.27 |
| **140** | 15.3 | 130 | NA | 43.45 | 17.05 | 14.95 | 1.14 | 15.10 |
| **141** | 22.3 | 411 | 60.44 | 46.94 | 22.43 | 20.93 | 1.07 | 28.00 |
| **142** | 15.6 | 137 | 67.95 | 36.90 | 15.88 | 14.68 | 1.08 | 15.70 |
| **143** | 18.4 | 223 | 64.17 | 40.78 | 21.67 | 19.57 | 1.11 | 22.30 |
| **144** | 17 | 181 | 57.43 | 43.25 | 22.10 | 17.93 | 1.23 | 17.67 |
| **145** | 16.6 | 205 | 65.39 | 40.77 | 19.43 | 16.88 | 1.15 | 20.23 |
| **146** | 16.3 | 146 | 57.01 | 41.13 | 17.50 | 15.80 | 1.11 | 16.70 |
| **147** | 17.4 | 202 | 63.97 | 46.77 | 21.13 | 19.97 | 1.06 | 20.27 |
| **148** | 15.2 | 115 | 60.19 | 40.42 | NA | NA | NA | 16.20 |
| **149** | 21.1 | 401 | 61.99 | 39.33 | 23.43 | 22.87 | 1.02 | 22.23 |
| **150** | 22 | 447 | 66.83 | 43.99 | 25.60 | 21.40 | 1.20 | 29.03 |
| **151** | 23.2 | 616 | 70.73 | 45.72 | 25.63 | 22.60 | 1.13 | 31.67 |
| **152** | 20.4 | 317 | 68.31 | 41.77 | 19.93 | 19.53 | 1.02 | 24.18 |
| **153** | 19 | 262 | 65.35 | 49.21 | 20.23 | 19.33 | 1.05 | 24.47 |
| **154** | 18.3 | 279 | 58.24 | 37.15 | 21.80 | 20.18 | 1.08 | 21.77 |
| **155** | 23.2 | 537 | NA | 50.12 | 24.57 | 22.73 | 1.08 | 31.37 |
| **156** | 17.5 | 182 | 67.72 | 39.73 | 17.93 | 15.02 | 1.19 | 17.05 |
| **157** | 18 | 205 | 67.13 | 39.71 | 19.03 | 16.50 | 1.15 | 20.07 |
| **158** | 16 | 140 | 57.57 | 40.35 | 17.05 | NA | NA | 16.18 |
| **159** | 20.6 | 322 | 67.90 | 50.28 | 19.97 | 19.27 | 1.04 | 26.47 |
| **160** | 22.5 | 427 | 69.53 | 45.12 | 24.03 | 21.30 | 1.13 | 30.30 |
| **161** | 17.2 | 218 | 61.91 | 46.79 | 19.13 | 15.72 | 1.22 | 20.47 |
| **162** | 18.3 | 217 | 56.56 | 41.19 | 21.27 | 19.67 | 1.08 | 21.43 |
| **163** | 18 | 237 | 61.91 | 40.12 | 22.30 | 17.93 | 1.24 | 22.83 |
| **164** | 17.3 | 210 | 62.95 | 39.24 | 19.90 | 18.62 | 1.07 | 21.80 |
| **165** | 19.1 | 242 | 61.40 | 49.96 | 20.73 | 17.93 | 1.16 | 24.77 |
| **166** | 21.1 | 367 | 71.23 | 42.94 | 19.60 | 19.70 | 0.99 | 25.47 |
| **167** | 16 | 121 | 65.92 | 42.18 | 16.13 | 12.17 | 1.33 | 17.37 |
| **168** | 16 | 150 | 56.84 | 40.29 | 18.73 | 16.17 | 1.16 | 17.67 |
| **169** | 23 | 567 | 62.11 | 53.82 | 23.33 | 22.80 | 1.02 | 31.13 |
| **170** | 17 | 175 | 59.53 | 46.45 | 19.52 | 16.60 | 1.18 | 19.58 |
| **171** | 20.3 | 329 | 64.46 | 50.61 | 20.83 | 19.67 | 1.06 | 25.67 |
| **172** | 21.4 | 469 | 58.68 | 43.71 | NA | NA | NA | 29.90 |
| **173** | 20.1 | 375 | 62.99 | 45.34 | 22.63 | 21.60 | 1.05 | 27.20 |
| **174** | 22.6 | 553 | 69.02 | 44.49 | 22.73 | 23.77 | 0.96 | 29.57 |
| **175** | 16.2 | 167 | 74.31 | 44.35 | 18.33 | NA | NA | 17.53 |
| **176** | 16.2 | 170 | 65.98 | 41.13 | 18.05 | 16.65 | 1.08 | 19.20 |
| **177** | 17.6 | 224 | 63.07 | 39.34 | 20.13 | 18.30 | 1.10 | 19.30 |
| **178** | 16.6 | 156 | 70.00 | 42.20 | 17.70 | 15.00 | 1.18 | 18.80 |
| **179** | 23.3 | 560 | 58.91 | 39.28 | 26.07 | 23.33 | 1.12 | 30.23 |
| **180** | 15.5 | 175 | 61.93 | 42.74 | 18.23 | 16.47 | 1.11 | 16.62 |
| **181** | 18.5 | 272 | 64.36 | 41.85 | 22.50 | 20.47 | 1.10 | 22.17 |
| **182** | 20.1 | 308 | NA | 41.21 | 23.10 | 19.70 | 1.17 | 24.67 |
| **183** | 21.1 | 468 | 66.79 | 44.84 | 22.93 | 18.93 | 1.21 | 26.67 |
| **184** | 16.1 | 158 | 60.09 | 42.82 | 19.28 | 16.23 | 1.19 | 17.67 |
| **185** | 23.1 | 516 | 72.05 | 48.51 | 23.47 | 21.80 | 1.08 | 30.13 |
| **186** | 19.2 | 269 | 59.86 | 40.15 | 21.63 | 19.90 | 1.09 | 24.67 |
| **187** | 22.4 | 547 | 62.07 | 50.76 | 24.80 | 22.47 | 1.10 | 31.47 |
| **188** | 16.6 | 175 | 56.72 | 39.10 | 18.73 | 15.75 | 1.19 | 18.88 |
| **189** | 15.4 | 145 | 56.41 | 37.26 | 18.38 | NA | NA | 17.18 |
| **190** | 18.1 | 223 | 65.94 | 44.39 | 19.17 | 18.37 | 1.04 | 19.37 |
| **191** | 17.2 | 234 | 55.90 | 37.27 | 18.73 | NA | NA | 18.93 |
| **192** | 17.4 | 202 | 61.10 | 46.58 | 18.32 | 16.27 | 1.13 | 18.90 |
| **193** | 18.4 | 244 | 73.89 | 44.74 | 20.53 | 18.53 | 1.11 | 22.80 |
| **194** | 19.1 | 229 | 65.69 | 44.44 | 19.77 | 16.43 | 1.20 | 22.90 |
| **195** | 22 | 479 | 64.18 | 38.89 | 25.07 | 24.80 | 1.01 | 28.10 |
| **196** | 17.3 | 198 | 55.65 | 38.33 | 19.68 | NA | NA | 21.30 |
| **197** | 18.2 | 232 | 63.08 | 43.22 | 20.20 | 19.53 | 1.03 | 22.13 |
| **198** | 15 | 130 | 56.24 | 43.17 | 16.87 | NA | NA | 16.03 |
| **199** | 22.4 | 444 | 66.99 | 43.84 | 22.80 | 20.23 | 1.13 | 28.37 |
| **200** | 21.3 | 409 | 65.73 | 49.97 | 23.57 | 24.13 | 0.98 | 27.13 |
| **201** | 20.1 | 335 | 67.41 | 45.47 | 21.67 | 19.43 | 1.11 | 24.13 |
| **202** | 23.1 | 578 | 65.44 | 43.09 | 24.73 | 21.67 | 1.14 | 30.17 |
| **203** | 16.3 | 158 | 57.17 | 44.00 | 16.83 | 15.62 | 1.08 | 17.75 |
| **204** | 15.6 | 134 | 62.41 | 38.94 | 15.68 | NA | NA | 15.37 |
| **205** | 20.4 | 328 | 63.15 | 41.35 | 20.70 | 19.50 | 1.06 | 25.40 |
| **206** | 19.1 | 291 | 61.44 | 38.72 | 19.77 | 18.87 | 1.05 | 19.60 |
| **207** | 23.4 | 543 | 64.53 | 42.37 | 24.42 | 23.17 | 1.05 | 29.95 |
| **208** | 15.3 | 126 | 64.97 | 43.01 | 17.38 | NA | NA | 16.22 |
| **209** | 23.2 | 480 | 53.69 | 42.15 | 26.23 | 24.93 | 1.05 | 30.07 |
| **210** | 16.3 | 151 | 67.57 | 42.19 | 18.80 | 15.68 | 1.20 | 17.50 |
| **211** | 22.6 | 483 | NA | 47.45 | 23.50 | 20.47 | 1.15 | 28.90 |
| **212** | 21.4 | 363 | NA | 38.50 | 21.43 | 20.50 | 1.05 | 20.40 |
| **213** | 20.6 | 325 | 61.00 | 42.31 | 22.15 | 18.70 | 1.18 | 25.60 |
| **214** | 20.4 | 333 | 58.88 | 45.37 | 23.43 | 20.90 | 1.12 | 25.83 |
| **215** | 21.4 | 365 | 75.82 | 41.68 | 21.43 | 20.53 | 1.04 | 26.33 |
| **216** | 15.2 | 120 | 69.79 | 33.94 | 17.43 | 16.30 | 1.07 | 15.40 |
| **217** | 17.6 | 229 | 49.53 | 37.39 | 19.18 | 16.83 | 1.14 | 20.05 |
| **218** | 17 | 188 | 65.02 | 45.68 | 18.57 | 16.27 | 1.14 | 20.57 |
| **219** | 15.3 | 128 | 65.69 | 47.12 | 18.07 | 15.17 | 1.19 | 15.67 |
| **220** | 21.4 | 461 | 71.94 | 50.27 | 23.73 | 23.30 | 1.02 | 28.73 |
| **221** | 16.1 | 117 | 64.13 | 36.88 | 19.23 | NA | NA | 15.02 |
| **222** | 21.1 | 410 | 59.67 | NA | 23.90 | 23.33 | 1.02 | 28.27 |
| **223** | 22.4 | 484 | 68.76 | 44.35 | 25.53 | 23.00 | 1.11 | 31.53 |
| **224** | 23.3 | 540 | NA | 39.44 | NA | 24.77 | NA | 28.93 |
| **225** | 23 | 470 | 64.81 | 43.24 | 25.23 | 22.83 | 1.11 | 30.37 |
| **226** | 21.5 | 334 | 60.74 | 38.41 | 22.37 | 21.20 | 1.06 | 26.30 |
| **227** | 18.6 | 249 | NA | 40.09 | 20.28 | 18.00 | 1.13 | 21.53 |
| **228** | 16.5 | 162 | 58.36 | 45.98 | NA | 15.90 | NA | 17.37 |
| **229** | 17.2 | 194 | 64.44 | 45.72 | 19.45 | 17.32 | 1.12 | 18.55 |
| **230** | 17.2 | 191 | 56.27 | 39.50 | 18.57 | 16.70 | 1.11 | 18.30 |
| **231** | 19.5 | 311 | NA | NA | 15.67 | 20.57 | 1.08 | 24.07 |
| **232** | 16.1 | 123 | 54.08 | 43.99 | 18.87 | NA | NA | 16.60 |
| **233** | 17.5 | 233 | 55.70 | 42.60 | 17.80 | 16.97 | 1.05 | 20.10 |
| **234** | 20.2 | 318 | 58.17 | 44.20 | 21.00 | 20.37 | 1.03 | 25.20 |
| **235** | 16.1 | 171 | 64.14 | 42.64 | 19.22 | 17.50 | 1.10 | 19.62 |
| **236** | 22.1 | 388 | 63.67 | 48.00 | 22.07 | 20.40 | 1.08 | 27.00 |
| **237** | 15 | 115 | 74.90 | 36.99 | 19.18 | NA | NA | 15.87 |
| **238** | 17 | 157 | 63.01 | 36.81 | 18.88 | 16.77 | 1.13 | 16.70 |
| **239** | 21.3 | 476 | 66.58 | 39.09 | 23.90 | 23.00 | 1.04 | 27.13 |
| **240** | 16.3 | 152 | 58.96 | 48.97 | 18.57 | 15.93 | 1.17 | 18.72 |
| **241** | 16.5 | 155 | 68.00 | 42.22 | 18.07 | 16.23 | 1.11 | 18.27 |
| **242** | 16.3 | 195 | 66.87 | 44.08 | 19.08 | NA | NA | 18.42 |
| **243** | 21 | 375 | 60.80 | 47.44 | 23.97 | 21.77 | 1.10 | 27.00 |
| **244** | 20.4 | 291 | NA | 41.63 | 21.60 | 19.27 | 1.12 | 25.43 |
| **245** | 17.1 | 205 | 71.82 | 46.94 | 17.38 | NA | NA | 18.43 |
| **246** | 16.2 | 155 | 67.49 | 40.23 | 18.05 | NA | NA | 17.90 |
| **247** | 16.2 | 163 | 63.58 | 39.78 | 19.30 | NA | NA | 16.85 |
| **248** | 20.5 | 418 | 73.08 | NA | 23.00 | 21.30 | 1.08 | 27.80 |
| **249** | 15.5 | 130 | 62.80 | 42.88 | 18.90 | 16.40 | 1.15 | 16.65 |
| **250** | 16.4 | 151 | 67.05 | 46.07 | 17.37 | 15.72 | 1.10 | 16.95 |
| **251** | 17 | 183 | 65.11 | 43.33 | 18.50 | 16.93 | 1.09 | 20.68 |
| **252** | 19.4 | 273 | 63.36 | 46.19 | 20.43 | 18.55 | 1.10 | 23.92 |
| **253** | 19.5 | 323 | 62.39 | 44.72 | 21.57 | 20.37 | 1.06 | 27.17 |
| **254** | 17.3 | 184 | 66.29 | 47.00 | 19.57 | 15.72 | 1.24 | 20.68 |
| **255** | 17.6 | 180 | 55.21 | 39.93 | 19.92 | 17.85 | 1.12 | 18.82 |
| **256** | 20 | 297 | 58.36 | 45.66 | 20.28 | 18.80 | 1.08 | 25.10 |
| **257** | 17.5 | 205 | 66.94 | 44.75 | 21.17 | 18.20 | 1.16 | 21.73 |
| **258** | 17 | 175 | 54.26 | 43.37 | 17.85 | 15.20 | 1.17 | 19.02 |
| **259** | 21 | 405 | 62.03 | 51.02 | 23.37 | 21.87 | 1.07 | 28.60 |
| **260** | 16.4 | 153 | 56.01 | 40.14 | 18.88 | 16.97 | 1.11 | 17.83 |
| **261** | 21.6 | 491 | 56.70 | 48.00 | 23.33 | 21.50 | 1.09 | 30.43 |
| **262** | 18 | 215 | 69.07 | 48.75 | 21.07 | 19.37 | 1.09 | 20.10 |
| **263** | 19.4 | 231 | 61.26 | 41.04 | 19.10 | 18.43 | 1.04 | 22.40 |
| **264** | 19.2 | 288 | 54.68 | 44.96 | 20.33 | 17.80 | 1.14 | 23.57 |
| **265** | 23.1 | 562 | 66.19 | 46.54 | 23.47 | 22.10 | 1.06 | 31.10 |
| **266** | 21.2 | 441 | NA | 42.18 | 24.13 | 20.07 | 1.20 | 28.10 |
| **267** | 22.2 | 490 | 66.13 | 50.28 | 26.47 | 22.30 | 1.19 | 30.40 |
| **268** | 18.2 | 239 | 65.76 | 45.42 | 21.17 | 17.30 | 1.22 | 23.00 |
| **269** | 21.4 | 417 | 67.38 | 43.16 | 21.97 | 19.77 | 1.11 | 29.00 |
| **270** | 23.1 | 564 | 66.69 | 47.37 | 24.90 | 22.60 | 1.10 | 31.47 |
| **271** | 23.2 | 490 | 72.76 | 45.07 | 23.17 | 20.97 | 1.10 | 29.47 |
| **272** | 20 | 267 | 61.82 | 40.89 | 20.40 | 18.63 | 1.09 | 23.60 |
| **273** | 21 | 360 | 56.01 | 37.38 | 19.97 | 17.20 | 1.16 | 22.70 |
| **274** | 18.5 | 215 | 57.90 | 38.28 | 19.63 | 18.87 | 1.04 | 20.50 |
| **275** | 23.6 | 551 | 69.09 | 42.68 | 24.67 | 24.27 | 1.02 | 33.70 |
| **276** | 19.2 | 323 | 64.96 | 42.95 | 21.17 | 19.63 | 1.08 | 26.43 |
| **277** | 18.5 | 241 | 64.83 | 43.94 | 20.33 | 15.97 | 1.27 | 22.67 |
| **278** | 21.3 | 424 | 64.53 | 39.90 | 23.93 | 21.37 | 1.12 | 24.93 |
| **279** | 20.2 | 325 | NA | 43.16 | 20.77 | 18.50 | 1.12 | 24.30 |
| **280** | 21.3 | 374 | 63.54 | 43.01 | 21.63 | 20.03 | 1.08 | 26.13 |
| **281** | 21.1 | 403 | 53.68 | 42.59 | 22.23 | 20.87 | 1.07 | 26.37 |
| **282** | 18.3 | 270 | 64.32 | 41.72 | 20.10 | 20.10 | 1.00 | 25.63 |
| **283** | 20.6 | 365 | 72.53 | 45.36 | 22.80 | 20.40 | 1.12 | 25.50 |
| **284** | 22.2 | 456 | 60.92 | 36.66 | 22.93 | 21.80 | 1.05 | 30.50 |
| **285** | 21.2 | 379 | 63.94 | 52.48 | 22.17 | 21.33 | 1.04 | 24.67 |
| **286** | 21 | 354 | 59.66 | 44.56 | 20.40 | 18.80 | 1.09 | 22.07 |
| **287** | 21 | 323 | 62.35 | 43.03 | 21.60 | 19.50 | 1.11 | NA |
| **288** | 21 | 401 | 67.67 | 49.59 | 22.03 | 20.47 | 1.08 | 28.43 |
| **289** | 21 | 383 | 52.50 | 40.24 | 23.97 | 22.53 | 1.06 | 26.93 |
| **290** | 22.3 | 497 | 63.82 | 49.22 | 24.87 | 21.93 | 1.13 | 31.83 |
| **291** | 16 | 143 | 66.76 | 38.80 | 16.68 | NA | NA | 15.55 |

NA : no data available due to inability to obtain proper images
